# West Nile Virus Circulation in Mosquitoes in Greece (2010–2013)

**DOI:** 10.1155/2016/2450682

**Published:** 2016-05-12

**Authors:** Eleni Patsoula, Annita Vakali, Georgios Balatsos, Danai Pervanidou, Stavroula Beleri, Nikolaos Tegos, Agoritsa Baka, Gregory Spanakos, Theano Georgakopoulou, Persefoni Tserkezou, Wim Van Bortel, Herve Zeller, Panagiotis Menounos, Jenny Kremastinou, Christos Hadjichristodoulou

**Affiliations:** ^1^Department of Parasitology, Entomology and Tropical Diseases, National School of Public Health, 11521 Athens, Greece; ^2^Hellenic Center for Disease Control and Prevention, 15123 Athens, Greece; ^3^School of Medicine, Department of Hygiene and Epidemiology, University of Thessaly, 41500 Larissa, Greece; ^4^European Centre for Disease Prevention and Control, Stockholm, 17165 Solna, Sweden; ^5^Molecular Biology Laboratory, 401 General Military Hospital of Athens, 11525 Athens, Greece

## Abstract

*Background of the Study*. Following a large West Nile virus (WNV) epidemic in Northern Greece in 2010, an active mosquito surveillance system was implemented, for a 3-year period (2011, 2012, and 2013).* Description of the Study Site and Methodology*. Using mainly CO_2_ mosquito traps, mosquito collections were performed. Samples were pooled by date of collection, location, and species and examined for the presence of WNV.* Results*. Positive pools were detected in different areas of the country. In 2010, MIR and MLE values of 1.92 (95% CI: 0.00–4.57) and 2.30 (95% CI: 0.38–7.49) were calculated for the Serres Regional Unit in Central Macedonia Region. In 2011, the highest MIR value of 3.71(95% CI: 1.52–5.91) was recorded in the Regions of Central Greece and Thessaly. In 2012, MIR and MLE values for the whole country were 2.03 (95% CI: 1.73–2.33) and 2.15 (95% CI: 1.86–2.48), respectively, for* Cx. pipiens*. In 2013, in the Regional Unit of Attica, the one outbreak epicenter, MIR and MLE values for* Cx. pipiens* were 10.75 (95% CI: 7.52–13.99) and 15.76 (95% CI: 11.66–20.65), respectively.* Significance of Results/Conclusions*. The contribution of a mosquito-based surveillance system targeting WNV transmission is highlighted through the obtained data, as in most regions positive mosquito pools were detected prior to the date of symptom onset of human cases. Dissemination of the results on time to Public Health Authorities resulted in planning and application of public health interventions in local level.

## 1. Introduction

WNV is an RNA virus within the Flaviviridae family, a member of the Japanese encephalitis virus serocomplex [1]. Mosquitoes of the genus* Culex* mainly and to a lesser extent* Aedes* spp. and* Ochlerotatus* spp. act as vectors, while several bird species act as amplifying hosts [2–4]. Horses, humans, and other mammals are also enrolled, acting as incidental or dead-end hosts.

Infection from West Nile virus (WNV) is an important public health issue for many European countries including Greece since 2010. WNV circulation has been recorded in several countries across the European continent, both in Central Europe and in the Mediterranean basin [5–9].

During the summer of 2010, Greece underwent the second largest WNV infection outbreak in Europe, after the outbreak of 1996 in Romania [10], with a total of 262 clinical human cases and 35 deaths. The majority of the cases were observed close to four rivers forming a large Delta, a major Mediterranean wetland close to Thessaloniki in Central Macedonia [11]. In the past, WNV serological surveys performed in Greece [12, 13] gave a first indication regarding WNV circulation.

Two main WNV genetic lineages have been reported [14, 15]. Lineage 1 has been found in Europe, Africa, Middle East, America, Australia, and India, whereas lineage 2 has been initially recorded in sub-Saharan Africa and Madagascar.

WNV strains were initially considered to differ considerably in terms of virulence and neuroinvasiveness and lineage 1 strains were considered as the only pathogenic forms of the virus. Recent studies, however, indicated that virulent and neuroinvasive strains of lineage 2 WNV exist in Europe and demonstrated similar ratios of infection to clinical disease to those found previously for WNV lineage 1 [16]. They were also detected in Greece in 2010 [17, 18] and in Italy in September 2011 [19]. Furthermore, there is evidence that at least two lineage 2 strains have been introduced and established in Europe that can cause severe disease to bird, horses, and human population [20].

In August 10, 2010, a pool of 50* Cx. pipiens* mosquitoes from the area of Nea Santa, a village located 30 km northeast of Thessaloniki in Central Macedonia, was found to be positive for WNV RNA by reverse transcription-PCR, the same area where the first human encephalitis cases had been reported. The 146-nucleotide fragment of the nonstructural protein 5 (NS5) gene revealed that the virus belonged to lineage 2 and presented the highest homology to Hungarian and South African strains, differing from the respective Russian WNV lineage 2 strain [21]. WNV lineage 2 was also identified in wild birds in Central Macedonia, Greece, during the 2010 transmission period (June–October) from areas in close proximity to human cases [22].

WNV lineage 2 sequences (strain Nea Santa-Greece-2010) were obtained from blood donors, mosquitoes, and birds in 2010 and 2011 transmission periods [17, 21–25].

In the past, sporadic entomological studies had been performed in Greece from institutes and organizations, although central data collection and analysis were lacking. After the 2010 WNV infection outbreak, a continuous and systematic entomological surveillance program was implemented for the first time in the country. The Hellenic Center for Disease Control and Prevention (HCDCP) confronted the urgent situation in cooperation with international and national authorities, aiming to set up standards for a future national strategic plan. Since 2010, HCDCP organized working groups with national and local stakeholders, such as the Ministry of Health and Social Solidarity (MoH), the Ministry of Rural Development and Food (MinAgric), the Department of Parasitology, Entomology and Tropical Diseases (DPETD) of the National School of Public Health (NSPH), the Benaki Phytopathological Institute (BPI), University Medical Schools, and local administration authorities, in order to manage the situation and to ensure the effective implementation of actions.

In 2010 a strategic framework for an integrated entomological surveillance program was proposed and the following points to be addressed were raised:

- Impact of current vector control strategies in terms of mosquito populations and potentially human epidemiology.
- Better understanding of species composition in affected and nonaffected villages and the transmission in rural versus urban sites.
- Reviewing of entomological situation in other parts of Greece.
- Impact of climate on mosquito abundance, any historical data that can be used.
- Better understanding of the transmission cycle and bird-mosquito-human interface.
- Understanding viral survival during the winter in mosquito populations [26].

In the context of this framework, entomological surveillance activities were initiated in Greece in 2011. The entomological surveillance in 2010–2013 was funded by the HCDCP and the MALWEST project “Control of West Nile Virus and Malaria-Strengthening of Surveillance in the Greek territory,” funded by NSRF (National Strategic Reference Framework) 2007–2013 [27].

In this study, we analyzed the data from the 2010–2013 mosquito surveillance in Greece, regarding the collected mosquitoes examined in the DPETD of the NSPH. The current study and the results presented are part of the national surveillance entomological plan of the Public Health Authorities for the Greek territory and included the vast majority of the affected regions by the WNV outbreak. The objectives of the study were

- to quantify the intensity of the virus circulation in mosquitoes at regional level in each transmission period,
- to describe the geographical and temporal distribution of the infected mosquitoes for each transmission period,
- to identify the temporal relation of the virus circulation in mosquitoes with the detection of human cases in the same region for each transmission period,
- to initiate the creation of a baseline of historical data, according to the national plan regarding entomological surveillance, in order to evaluate the use of mosquito surveillance indicators for a human infection risk assessment and a timely identification of geographical areas “at risk” for local virus transmission, in order to timely inform targeted preventive interventions.

## 2. Materials and Methods

### 2.1. Entomological Surveys

In 2010, a small scale entomological study was performed in Central Macedonia, at the epicenter of the WNV infection outbreak. The entomological surveillance followed the outbreak and the report of the majority of human cases, which mainly occurred in mid-August, and was implemented at the end of the transmission period. Mosquito collections were performed in Central Macedonia, in the villages with reported human cases and in surrounding areas. Traps were placed, by the Serres Prefecture Center for Mosquito Abatement (SPCMA) and the private company Ecodevelopment, in 67 stations mainly in Central Macedonia Region (Regional Units of Thessaloniki, Pella, Imathia, Kilkis, and Serres), from the 10/8/2010 until the 28/9/2010, and all samples were examined in NSPH.

**Figure 1:**
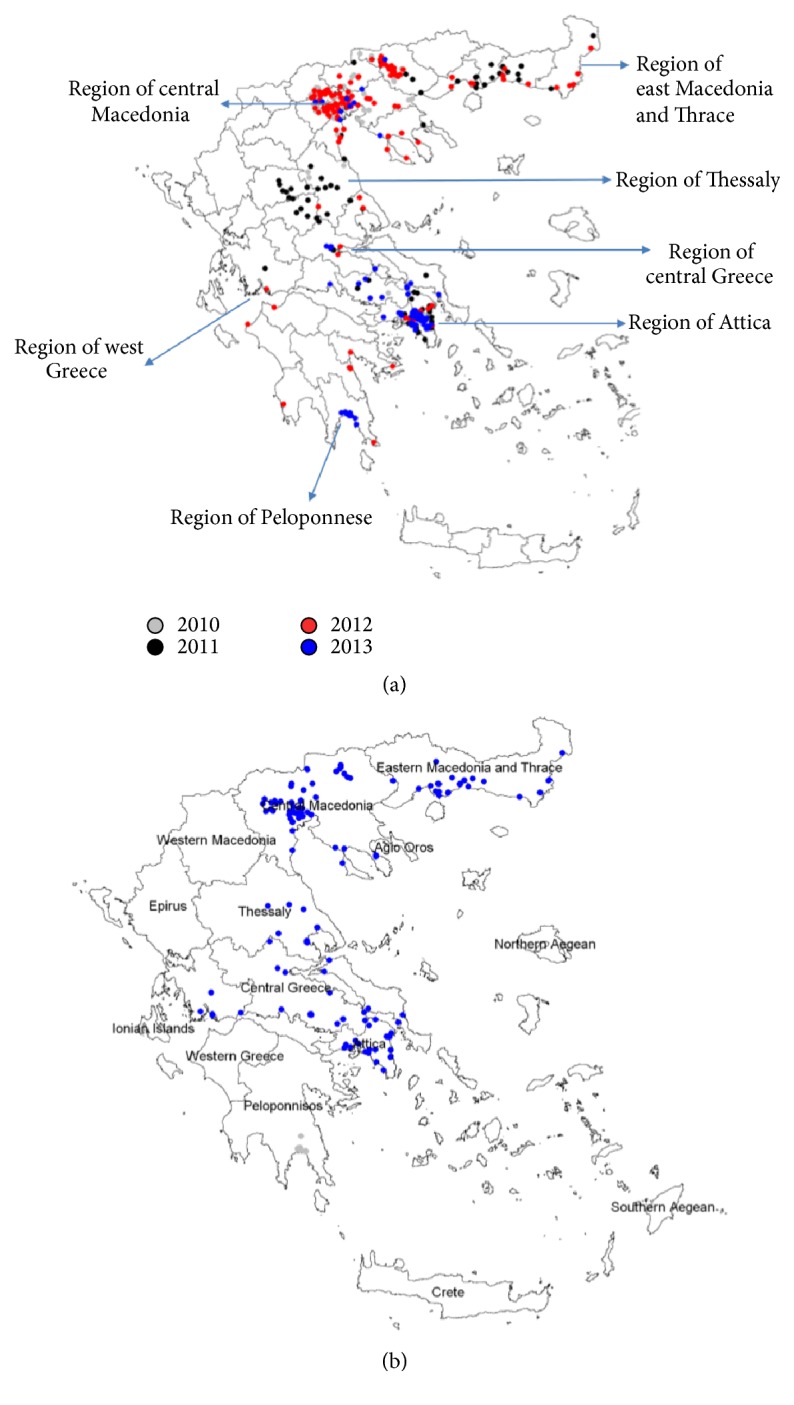
(a) Sampling points-mosquito traps with different symbols for each year, 2010–2013. (b) Traps with positive mosquito pools, 2010–2013 in blue.

In 2011, from 12/4 to 21/10, 140 geographically referenced (longitude and latitude) sampling stations operated in different parts of Greece. The protocol for field studies was approved by the Board HCDCP, supervised by the MoH. All regional and local authorities involved were aware of the surveillance project. No specific permit was required for trapping in the public areas. Verbal consent was provided by household owners in the private areas. The field studies did not involve endangered or protected species. The initial study design included the 2010 affected areas, that is, the Regions of Central Macedonia and East Macedonia and Thrace. During the 2011 study period, the mosquito surveillance was extended in the Regions of Central Greece, Attica, and Thessaly, following the occurrence of human cases, as well as in the Peloponnese Region (Figure 1(a)). Sixty of the stations were in Central Macedonia, the most affected region in 2010. Traps were placed by two private companies, subcontractors specialized in environmental applications, Ecodevelopment and Bioapplications, and by the Serres Prefecture Center for Mosquito Abatement with all samples being examined in NSPH. These sampling stations operated in a bimonthly or weekly frequency.

In 2012, from 10/5 to 30/10, and under the coordination of the MALWEST project [28], NSPH received samples from 209 fixed stations. Sampling stations were added as the virus was “moving” from North to South. These sampling stations operated, in a bimonthly frequency, in the Regions of Central Macedonia, East Macedonia, and Thrace, Thessaly, and Central Greece, including Attica Region, and were placed by the private companies Ecodevelopment, Bioapplications, and Axiven-Apolymantiki solutions and by the Serres Prefecture Center for Mosquito Abatement.

In 2013, due to the economic crisis in the country, a shorter scale active mosquito surveillance program was implemented within the framework of MALWEST project, a collaboration among the University of Thessaly, HCDCP, NSPH, BPI, Medical School of Athens, and three private constructor companies which implemented vector control programs funded by the prefectures. The sampling stations were located mainly in the Region of Attica, where traps were placed in locations with a higher risk of WNV transmission (reported human cases over the previous years, large breeding sites of* Culex* spp.), including Central Greece, Central Macedonia and Peloponnese, as well as in the Regional Unit of Dodecanese. In the context of these actions, mosquitoes were sent to NSPH, every 15 days.

The types of mosquito traps placed each year included CDC light traps with CO_2_ and to a lesser extent in 2013 BG-Sentinel/Mosquitaire and triple traps were also used. Traps were placed at dusk, from 6:00 pm, and were collected the following morning, approximately at 9:00 am. Mosquitoes were pooled according to date, location, and species, with a minimum of two and a maximum number of 200 individuals per pool. The whole trap was analyzed for the presence of WNV RNA. In the case that 2-3 mosquitoes of the same species were collected they were identified as one pool, whereas when a large number of mosquitoes were collected, they were divided in several pools of ≤200 mosquitoes, in accordance with several other studies [29–32]. The pooled mosquitoes were stored and frozen at −80°C.

### 2.2. Characteristic of the Study Areas

The study areas in the Region of Central Macedonia and Thrace are characterized by humidity, high temperatures, and increased rainfall during summer and early autumn. During winter temperatures vary between 5 and 10 degrees Celsius near the coasts and 0 and 5 over mainland areas, while in August the mean maximum temperature lies in the range of 29.0 and 35.0 degrees Celsius. River deltas, rice fields, and irrigated plants are present. In the Regions of Thessaly, Central Greece, and Peloponnese the climate condition is typically Mediterranean. Most common crops include cereals, vineyards, and olives and cultivation is spread in floodable areas near rivers. The costal part of the region is characterized by pinewood and typical Mediterranean vegetation. All the monitored areas are densely populated and characterized by the presence of villages, towns, and urban areas. The climate and geographical conditions favor the proliferation of mosquito populations (Hellenic National Meteorological Service, available at http://www.hnms.gr/hnms/english/climatology/climatology_html).

### 2.3. Mosquito Identification

All mosquitoes sent to the DPETD in NSPH were systematically identified to species level in the Laboratory of Medical Entomology, using morphological characteristics [33, 34].* Cx. pipiens* and* Oc. caspius* mosquitoes were tested for WNV. In 2013,* Aedes cretinus* (*Aedes (Stegomyia) cretinus* Edwards 1921 (Diptera: Culicidae)) and* Aedes albopictus* (*Aedes (Stegomyia) albopictus *(Skuse) 1895 (Diptera: Culicidae)) were also examined for WNV.

### 2.4. RNA Extraction-PCR

In a 13 mL tube, two 0.35 gr and 4.5 mm diameter stainless steel round balls (ASG, ActionSportGames, A/S, Humlebaek, Denmark) were added in case the mosquito pool included less than 50 individuals and 3-4 round balls for mosquito pools of 100 or more individuals. Different amounts of PBS were added in each tube according to the number of stored mosquitoes (0.5 mL until 30 specimens, 1 mL from 31 to 60 specimens, and 1.5–2 mL from 61 to 100 or more specimens). Furthermore, 2 *μ*L of Murine Rnase inhibitor (New England Biolabs, UK) was added in each tube.

Samples were vortexed for 1-2 mins in a vortex mixer and then centrifuged at 4,000 ×g for 3 mins. 200 *μ*L of the supernatant was collected for RNA extraction. During 2011, RNA isolation was performed using the Trizol LS reagent, according to manufacturer recommendations. The next step involved the addition of 600 *μ*L of Trizol LS Reagent (Invitrogen, Life Technologies) and samples were incubated in room temperature for 10 mins. Chloroform (Scharlab SL, Spain) was added (160 *μ*L); samples were mixed by hand and centrifuged for 10 mins in 14,000 rpm. The supernatant was collected (160 *μ*L) and placed in a fresh tube, an equal volume of isopropanol was added, and samples were placed in −20°C for 3 mins. They were subsequently centrifuged for 10 mins in 18,000 rpm. The supernatant was removed in a fresh tube and 500 *μ*L of 80% w/w ethanol (Scharlab SL, Spain) was added. Samples were centrifuged for 10 mins in 18,000 rpm and the supernatant was transferred in a fresh Eppendorf tube. Finally, samples were left for 10 mins at room temperature and 20 *μ*L of preheated UltraPure DNAse and RNAse free-water (Biochrom AG, Germany) was added.

From the summer of 2012 onwards, given that the number of tested pools was rising, a decision to move to an automated RNA extraction method was made, in order to decrease hands-on time and increase sample throughput, an essential prerequisite for a surveillance program, where the response time has to be minimal [35]. The Maxwell 16 Automated Nucleic Acid extraction system (Promega, Madison, WI), involving automated operation, guanidine-thiocyanate lysis, paramagnetic bead-based RNA purification, and enzymatic DNA elimination, was used for RNA extraction of high quality and yield from mosquito pools along with the Maxwell 16 LEV Simple RNA Tissue kit. The Maxwell 16 system is capable of extracting relatively large quantities of intact RNA, reduces the possibility of technician error, and has little or no risk of cross-contamination [36, 37]. The mosquito samples were initially treated as described above, with the addition of stainless steel round balls, PBS, Murine Rnase inhibitor, then vortexed, and centrifuged. The supernatant collected was used for subsequent RNA extraction with the Maxwell 16 Automated Nucleic Acid extraction system.

All pools were analyzed using polymerase chain reaction protocols (PCRs): (1) traditional PCR, targeted to NS5 gene fragment, for the detection of* Flavivirus* genus according to Scaramozzino et al. [38], utilizing cDNA prepared with the Superscript II kit (Invitrogen, Life Technologies) according to manufacturers' instructions. This protocol was used for an initial* Flavivirus* screening and for sequencing purposes, (2) commercial real-time PCR, LightMix® Kit West Nile virus, TibMolBiol, Roche along with the LightCycler® 1.0 instrument for the years 2010 and 2011, (3) from the summer of 2012 onwards, the highly sensitive TaqMan real-time PCR protocol of Tang et al. [39], targeting a conserved 92 bp fragment of the 3′ noncoding region of WNV, detecting only WNV lineages 1 and 2, was utilized. This real-time PCR protocol has already been used in several similar studies [30, 31, 40, 41]. Additionally, for years 2013 and 2014 and for further verification of the results, all positive samples were subjected to a second real-time PCR protocol specific for WNV detection of Eiden et al., 2010 [42], and all the results were in perfect accordance.

As a positive control, an RNA sample of WNV was used. As negative controls, RNA samples from several arthropod-borne viruses such as Dengue and Chikungunya viruses were used.

### 2.5. Single-Stranded Conformation Polymorphism (SSCP)

15 *μ*L of PCR products was denatured and electrophoresed in a polyacrylamide gel (7.2% acrylamide, 0.22% bisacrylamide, 1.25x TBE, 0.1% ammonium persulfate, and 0.8−3% TEMED) for 25 hrs at 14°C and 420 V. Respective bands were visualized with silver stain [43].

### 2.6. DNA Sequencing

The PCR products of the* Flavivirus* genus specific seminested protocol of Scaramozzino et al. [38] were excised from a 2% agarose gel and DNA was isolated using the QIAquick Gel extraction kit (QIAGEN, Hilden, Germany), according to manufacturer's instructions. Sequencing was performed at the Department of Immunology and Histocompatibility, School of Medicine, University of Thessaly (Larissa, Greece), using primers employed in the inner nested PCR according to the manufacturer's instructions. Obtained sequences were initially employed to perform basic local alignment search tool (BLAST) in the GenBank library to confirm the specificity of positive reactions and to estimate the degree of identity of previously detected strains. The obtained sequences were aligned with available deposited GenBank sequences by Clustal Omega available at http://www.ebi.ac.uk/Tools/msa/clustalo/.

### 2.7. Infection Rates

Minimum infection rates (MIR) and maximum likelihood estimation rates (MLE) were calculated using the PooledInfRate program version 4.0 (available at http://www.cdc.gov/ncidod/dvbid/westnile/software.html). For each region included in the study areas, the respective MIR and MLE values were calculated per 1.000 mosquitoes tested [44, 45].

## 3. Results

### 3.1. Traps and Mosquito Samples

In 2010, the limited mosquito collection at the end of the transmission period resulted in collecting 6,597* Cx. pipiens* mosquitoes from August 8 until September 28, at the Regional Units of Thessaloniki, Pella, Imathia, Kilkis, and Serres in Central Macedonia Region, where human cases were previously detected. Out of the 110 pools tested, two were found positive for WNV in the Regional Unit of Serres, with both protocols (nested PCR and commercial real-time PCR) utilized. Location of mosquito traps and respective positive pools are presented in Figures 1(a) and 1(b).

In 2011, 78,224* Cx. pipiens* and* Oc. caspius* were collected from 12/4 to 21/10. Among them,* Cx. pipiens* mosquitoes (79% abundance) were identified in all monitoring stations. The remaining 21% of the* Oc. caspius* mosquitoes were collected from stations at the Region of East Macedonia and Thrace during August and September. 897 pools were tested and 71 of them in four distinct regions (Table 1) were positive for WNV with the combined molecular methodology used (nested PCR and commercial real-time PCR). All pools tested for WNV from April and May were found negative. The first positive samples were detected on 23/6/2011, in two pools from the Regional Unit of Thessaloniki, in Central Macedonia Region. The first human case of WNV infection from the Regional Unit of Thessaloniki was diagnosed on 29/7/2011. In the Regions of Thessaly and Central Greece, the detection of the first positive mosquito pools preceded the diagnosis of the human cases, by 13 and 36 days, respectively, whereas in the Region of East Macedonia and Thrace, with the first positive mosquito pool detected in July, no human cases were diagnosed in 2011. On the other hand, in the Regions of Attica and West Greece all mosquito pools tested were found negative for WNV although human WNV cases occurred. Additionally in the Region of Peloponnese all the mosquito pools tested were found negative for WNV, while no human WNV cases occurred (Table 2). Location of the mosquito traps and positive pools are presented in Figures 1(a) and 1(b). The majority of positive samples (80%) were detected in July and September.

In 2012, 99,856 mosquitoes were collected from April to October belonging to* Cx. pipiens* and* Oc. caspius* species.* Cx. pipiens* mosquitoes represented the highest abundant species (87.77%) and were collected in all monitoring stations. Overall 1,789 pools were tested, 207 of which were found positive for WNV in six regions (Table 3). 178 positive pools concerned* Cx. pipiens* mosquitoes.

The majority (124) of positive pools (59.9%) were detected in June and July. The Region of Attica and the Regional Unit of Thessaloniki presented with the most positive samples (18/182). The first human case of WNV infection in 2012 was diagnosed on 9/7/2012 in Attica Region, where the detection of the first positive mosquito pool had been preceded by >50 days. In four other regions (namely, the Regions of East Macedonia and Thrace, West Greece, Central Macedonia, and Central Greece) the detection of the first positive mosquito pools also preceded the diagnosis of the human cases (by 24, 66, 53, and 65 days, resp.), whereas in the Thessaly Region, with the first positive mosquito pool detected on 5/2012, no human cases were recorded in 2012. On the other hand, in the Regional Unit of Epirus all tested mosquito pools were found negative for WNV although human WNV cases occurred. In the Regions of North Aegean and Ionian Islands where human WNV cases occurred, no mosquito pools were tested for WNV. Additionally, in the Region of Peloponnese all tested mosquito pools were negative for WNV and no human WNV cases occurred (Table 2). Location of the mosquito traps and positive pools are presented in Figures 1(a) and 1(b).

In 2013, in all the Regional Units included in the short scale study, a total of 327 pools and 8,451 mosquitoes belonging to* Cx. pipiens, Oc. caspius, Ae. cretinus, and Ae. albopictus* were collected and tested, including 3,906 mosquitoes collected in Attica Region, from 5/2013 to 10/2013, through the active vector surveillance program implemented by the HCDCP, the MALWEST project, and the NSPH. In Attica Region,* Cx. pipiens* mosquitoes represented the highest percentage among the total collected mosquitoes (95.45%) and were present in all monitoring stations. The remaining 4.55% of the mosquitoes were also species described to have urban breeding sites besides the* Anopheles sacharovi* (*Anopheles (Anopheles) sacharovi* Favre 1903 (Diptera: Culicidae)), which was collected in a rural area. Forty-five mosquito pools in total, including 44* Cx. pipiens* pools, one* Ae. albopictus* pool, in Attica Region, and one* Cx. pipiens* pool collected from the Central Macedonia Region (Imathia Regional Unit), were found positive for WNV RNA. All* Oc. caspius* pools were negative.

The first positive mosquito pool in the Attica Region was detected on 28/5/2013, preceding the diagnosis of the first human case by 49 days. On the other hand, in the Regions of Ionian Islands and East Macedonia and Thrace, where human WNV cases occurred, no mosquito pools were tested for WNV. Additionally, in the Regions of Peloponnese and Central Greece all the mosquito pools tested negative for WNV and no human WNV cases occurred (Table 2).

The majority of the positive pools in Attica Region were detected in the Regional Unit of the Northern section of Athens, the human outbreak epicenter, from 28/5/2013 to 10/7/2013, the majority of them detected in June, 26 out of 45 (57.7%). The activity of the virus probably started before the mosquito surveillance actions, since four out of the first 11 pools collected within the period 27–31/5/2013 were found positive for WNV.

In the Imathia Regional Unit, one positive pool was detected 14 days after the diagnosis of the first human WNV case. On the other hand, in the Regions of Ionian Islands, East Macedonia and Thrace, and West Greece, where human WNV cases occurred, no mosquito pools were tested for WNV. Additionally, in the Regions of Peloponnese and Central Greece all tested mosquito pools were negative for WNV and no human WNV cases occurred (Table 2).

### 3.2. MIR

In 2011 MIR and MLE values were highest in Central Greece and Thessaly region (3.71 and 3.87, resp.). In 2012, the highest MIR of 14.98 (95% CI: 9.74–20.21) and MLE rate of 22.80 (95% CI: 17.18–31.39) were recorded in Central Greece in* Cx. pipiens* pools, followed by Attica with 7.65 (95% CI: 4.13–11.17) and MLE rate of 8.05 (95% CI: 5.08–12.24) (Table 4). In 2013, the MIR and MLE values in Attica further increased (to 10.75 and 15.67, resp.) (Table 4).

### 3.3. SSCP and Sequencing

In 2011, 71 positive pools were detected. The PCR products of nine positive samples were sequenced and respective WNV lineage 2 sequences were obtained and deposited in EMBL European Nucleotide Archive (accession numbers HE716844–HE716852). These sequences share 100% homology with the HQ537483 Nea Santa Greece/2010 WNV strain (nucleotides 9116–9282) detected in* Culex pipiens* mosquitoes in the summer of 2010 [17, 21], with the JN858070 West Nile virus isolate Italy/2011 [19] and the HM015884 West Nile virus strain goshawk Austria 361/10 (2009) NS5 gene [46].

**Figure 2:**
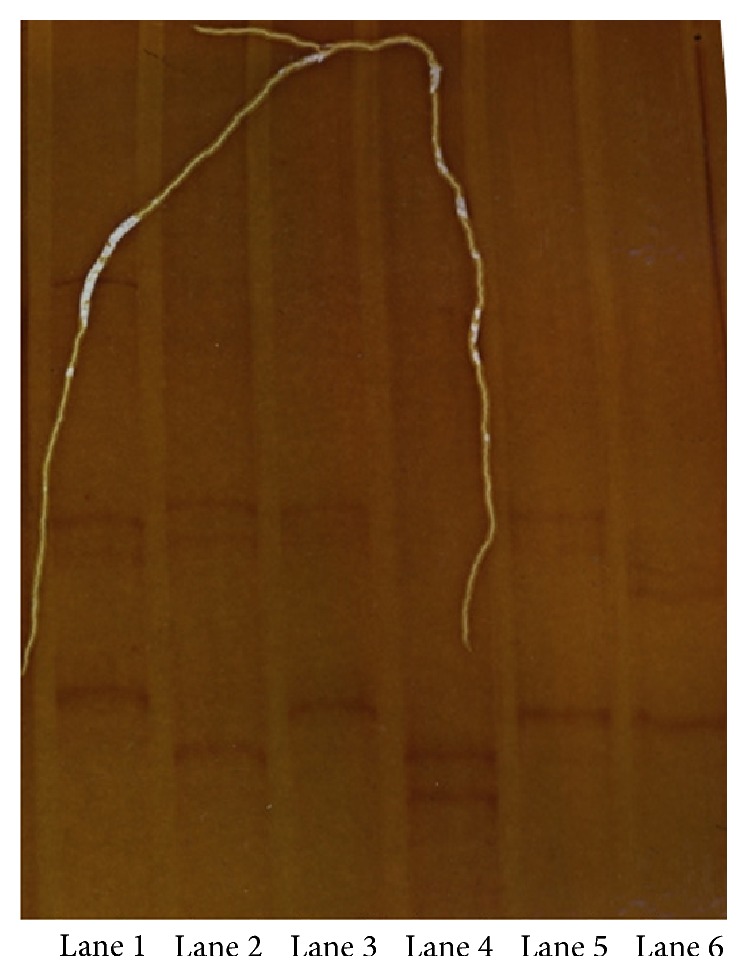
Single-strand conformational polymorphism profiles of the* Flavivirus* PCR products. Templates used with their EMBL code were Lane 1, HE716845, Lane 2, HF543929, Lane 3, HF543928, Lane 4, HF543926, Lane 5, HF543925, and Lane 6, HF543924. Lane 1, Lanes 3 and 5, Lane 2, Lane 4, and Lane 6 present five different SSCP profiles.

The remaining 62 positive samples were further analyzed with SSCP in order to minimize sequencing costs. Based on SSCP banding profiles of all samples, in total nine different profiles were detected. In Figure 2, we present SSCP results of six samples and five different profiles can be seen. In Lane 1 sample HE716845 presents a distinct profile, which corresponds to a nucleotide G in position 112 instead of an A in the other sequences, confirming the sensitivity of the SSCP methodology in detecting different genotypes. In Lanes 3 and 5 two samples that form a second SSCP profile are presented, and three more profiles in Lanes 2, 4, and 6 can be seen. The twelve sequences deposited to EMBL and HF543921–HF543932 belong to one of the five different profiles and share homology ranging from 98% to 100% on 167 positions with the previously deposited ones (HE716844–HE716852) and with the Greek 2010 strain (HQ537483) and were characterized as silent, as they did not produce any amino acid sequence variations. The four sequences with EMBL numbers HF543933–HF543936 corresponding to four different SSCP profiles were 164 bp long, and BLAST analysis revealed 98% identity with mosquito* Flavivirus* isolate FE-Cp NS5 gene from Italy. These four samples were excluded from the MIR/MLE calculation.

In 2012, a total of 20 positive samples from the Regional Units of Attiki, Fthiotida, Viotia, Magnisia, Larisa, Aetolia-Acarnania, Fokida, Euboea, Imathia, Chalkidiki, Evros, Xanthi, and Thessaloniki have been sequenced. In all cases WNV lineage 2 sequences were obtained, presenting 98 to 100% homology with HQ537483 Nea Santa Greece/2010 WNV strain. Thirteen sequences from different regional units were deposited in EMBL European Nucleotide Archive with accession numbers HG973476 to HG973488.

In 2013, sequencing verification was also performed on the first eight positive samples from Attica. WNV lineage 2 sequences were obtained with complete identity with the Nea Santa Greece/2010 WNV strain. Six sequences were deposited in EMBL European Nucleotide Archive, with accession numbers HG973489–HG973494.

## 4. Discussion

In the current study, we present all the available data from the four first years of the WNV transmission in Greece. This is the first attempt to design and implement an entomological study on a national level. A large amount of data is being presented separately for each transmission period. Previously, only sporadic entomological studies have been performed, lacking centralized data collection and analysis. During the preparation phase for the 2004 Olympic Games the NSPH conducted mosquito surveillance studies in areas close to Olympic venues [47]. After 2004, entomological surveys were performed by private companies under contracts with Prefectures or Municipalities concerning mostly mosquito control programs. The need for initiating mosquito surveillance actions emerged after recording of the first human cases of WNV in Greece in 2010, in order to monitor the occurrence of vector-borne diseases, gain a better knowledge of the mosquito populations by identifying potential vectors of arboviruses, and investigate arbovirus infection. Until that time, there was only limited information about mosquito species and their respective abundance across the country and specifically in the WNV outbreak epicenter.

For 2010, the study was limited, as mosquito collections were performed only occasionally and were not synchronous with the occurrence of human cases. It started and was carried out after the report of the majority of human cases. The mosquito collections were performed only occasionally, in an effort to follow the occurrence of human cases. At the same time emergency vector control interventions were implemented and the HCDCP performed extensive actions of informing the public in Central Macedonia regarding prevention and control measures. We took into consideration this limitation when designing the entomological surveillance programs for the following years and from 2011 onwards entomological studies were implemented during the whole transmission periods and larger geographical areas were covered as human cases also occurred in many different regions and not only in the Central Macedonia Region.

In 2011, the large majority of the traps were placed in the Region of Central Macedonia, the 2010 outbreak epicenter. However, the highest human disease incidences were recorded in the Regions of Central Greece and Thessaly, where only 99 mosquito pools from selected areas were collected in 2011, a limitation of the study. This was a weakness of the entomological surveillance system as initially it included a large number of mosquito traps in the Central Macedonia Region, the epicenter of 2010. However, in 2011 the virus “moved” towards southern parts of the country, Regions of Central Greece and Thessaly, a fact that could have not easily been predicted when designing the 2011 surveillance program, based on the 2010 epidemic. Nevertheless, the highest MIR and MLE values in 2011 were also recorded in the Regions of Central Greece and Thessaly, indicating that the quantification of the virus circulation in mosquitoes can likely provide evidence for the identification of areas “at risk” for human transmission. The calculated rates were in accordance with the ones recorded in Italy, other European countries, and the US [13, 31, 32, 48–51].

Detection of positive mosquito pools indicates circulation of the virus. In many regions, the entomological surveillance detected the circulation of the virus in an early phase, as the first positive mosquito pool preceded human cases. We have analyzed above the limitations and weaknesses of the entomological studies of each separate year and these observations can provide a useful tool for future improvements.

*Oc. caspius* is a mammophilic species occasionally found engorged with avian blood. It is not considered a very competent vector, with high infection rates.

Balenghien et al. [52], in order to identify the mosquito species able to sustain the transmission of WNV in southern France, also assessed among other species the vector competence of* Oc. caspius*. After incubation, the disseminated infection and transmission rates were 0.8% and 0 for* Oc. caspius*, as only two females orally exposed to WNV developed a disseminated infection. Therefore this species is considered as a poor WNV laboratory vector, despite its high densities. As it has been proven its role in WNV transmission may be minor. However,* Oc. caspius* densities can reach exceptional rates in one night, a fact that may compensate to some level for its low competence level for WNV. Its role in WNV transmission needs to be further studied.

The following year, 2012, the highest MIR and MLE rates were observed in the Region of Central Greece for* Cx. pipiens*. For this year, human cases in this region were limited; this finding could be attributed to the small number of adult mosquitoes captured and examined and the small pool size [13] or to underdiagnosis of human. On the other hand, high MIR and MLE rates were observed in Attica and East Macedonia and Thrace regions, which were the 2012 outbreak epicenters. The detection of the first positive mosquito pools preceded the onset of first human cases in the Regions of East Macedonia and Thrace, Attica, West Greece, Central Macedonia, and Central Greece (min 20 and max 65 days).

In 2013, in one WNV outbreak epicenter, in Attica Region, the first detected positive mosquito pool preceded the diagnosis of the human cases by 49 days, providing early detection of the virus circulation in the area. MIR and MLE estimated for the Region of Attica for* Cx. pipiens* mosquitoes were high, indicating the burden of the virus circulation in the epicenter.

Detection of the first positive mosquito pool indicating circulation of the virus preceded detection of first human case by 37 days in the Attica Region. Generally, in the present study, positive mosquito pools were detected mainly in regions and regional units where human cases were also recorded.

For the years 2011–2013 more extended studies were performed and positive mosquito pools detection preceded the diagnosed human cases in the 90% of the regions with both infected mosquitoes and humans detected. The collection of the first WNV-positive mosquito pool preceded the diagnosis of the first human case by an average of 46.6 days (range 13–66 days). For these three years, the detection of the WNV-positive mosquito pools was not followed, on a regional level, by human cases recorded during the same transmission period in two cases only, in the Region of East Macedonia and Thrace in 2011 and in the Thessaly Region in 2012.

It seems likely that the circulation of the virus in mosquitoes can be used as an early alert for the transmission of the virus in humans in, at least, a region level. Several studies have demonstrated a strong correlation between environmental factors and the incidence of WNV. The quantification of the virus circulation in mosquitoes can likely provide evidence for the identification of areas “at risk” for human transmission. The calculated rates in Greece were in accordance with the ones recorded in Italy, other European countries, and the USA [30, 32, 49–51].

Particularly since WNV is a seasonal disease, data obtained from the entomological surveillance can be used in order to increase the vigilance of the local authorities in intensifying the mosquito control measures as well as raise awareness among local physicians for the recognition and diagnosis of WNV disease. Laboratory results for mosquito identification and WNV detection are sent to HCDCP within 3-4 days. HCDCP timely disseminates these mosquito surveillance data to national, regional, and local stakeholders, as regional and local authorities fund, design, and implement the mosquito control programs. Whenever a positive mosquito pool is detected, the response plan is immediate and specific recommendations are made in order to perform special vector management applications in the areas indicated.

Limitations of the present study include the small scale program that was performed in 2010, due to lack of scheduled operational activities and the short scale active mosquito surveillance program implemented in 2013, due to economic crisis in the country. It is very important to state that the initial plan was to establish an entomological surveillance system with stable stations in order to monitor the regions affected and the majority of the regional units of Greece. However, the unstable financial situation of the country, the limited economical resources and the cutdown in funding of these programs forced the MoH to scale down the surveillance actions instead of completely eliminating them.

In general,* Cx. pipiens* was the main vector incriminated with the highest infection rates. The identification of the WNV lineage 2 (strain 2010 Nea Santa) revealed in all positive pools that were sequenced, from the Regions of Central Macedonia, East Macedonia and Thrace, Thessaly, Central Greece, and Attica, indicates that this lineage is well established in Greece.

## 5. Conclusions

Pathogen screening of mosquitoes is still in question in Europe, but in endemic areas like Greece, this can turn out to be a useful tool for predicting areas at risk of virus transmission to humans. In a country affected by emerging vector-borne diseases, such as the WNV infection, the mosquito surveillance is considered integral part of addressing this public health threat [53, 54]. Timely communication and information flow between the public health authorities at national and local level is of major importance for the creation and maintenance of a flexible and real-time response system, in order to implement prevention measures and efficiently limit the virus burden in a local level. It is noteworthy that, for 2015, no human cases were recorded in Greece. This study highlights the importance of entomological surveillance, as most of the mosquitoes collected were found to have vector competence. The availability of continuous data on mosquito populations, even in smaller scales, provides invaluable information for use in cases of an epidemic emergency. Maintenance of a surveillance system during the economic crisis is questionable. However, the active participation of the local authorities, for the next years, will provide more data that can be valuable for the design of a risk-based surveillance for the early detection of the occurrence of outbreaks of tropical mosquito-borne diseases.

## Figures and Tables

**Table 1:** Distribution of *Cx. pipiens* & *Oc. caspius* WNV pools in 2011.

Region	Identified mosquito species	Number of tested pools	Number of positive for WNV pools (% abundance)
Central Macedonia	*Cx. pipiens*	642	42 (6.5)
Central Greece & Thessaly	*Cx. pipiens*	99	11 (11.1)
East Macedonia	*Cx. pipiens*	83	7 (8.4)
Thrace	*Oc. caspius*	73	11 (15.1)
		Total: 897	Total: 71

**Table 2:** Summary of data regarding the date of onset of first human case, the date of diagnosis of first human case, the date of first mosquito pool tested, and the date of first positive mosquito pool, for years 2010–2013.

Region	2010				2011				2012				2013			
	Date of onset, first human case	Date of diagnosis, first human case	Date of first mosquito pool tested	Date of first positive mosquito pool	Date of onset, first human case	Date of diagnosis, first human case	Date of first mosquito pool tested	Date of first positive mosquito pool	Date of onset, first human case	Date of diagnosis, first human case	Date of first mosquito pool tested	Date of first positive mosquito pool	Date of onset, first human case	Date of diagnosis, first human case	Date of first mosquito pool tested	Date of first positive mosquito pool
East Macedonia & Thrace	5/10/2010	11/10/2010	—	—	—	—	25/6/2011	23/7/2011	30/7/2012	3/8/2012	24/5/2012	10/7/2012	16/7/2013	2/8/2013	—	—
Attica	—	—	—	—	25/7/2011	2/8/2011	22/6/2011	Negative	20/6/2012	9/7/2012	19/5/2012	19/5/2012	4/7/2013	16/7/2013	29/5/2013	28/5/2013
South Aegean	—	—	24/7/2013	Negative	1/9/2011	21/9/2011	—	—	—	—	—	—	—	—	—	—
North Aegean	—	—	—	—	—	—	—	—	17/7/2012	24/7/2012	—	—	—	—	—	—
West Greece	7/9/2010	27/9/2010	—	—	1/9/2011	7/10/2011	27/6/2011	Negative	25/7/2012	7/8/2012	16/5/2012	2/6/2012	12/8/2013	27/8/2013	—	—
West Macedonia	27/8/2010	1/9/2010	—	—	20/8/2011	30/8/2011	—	—	—	—	—	—	—	—	—	—
Epirus	—	—	—	—	—	—	—	—	14/9/2012	20/9/2012	20/6/2012	Negative	—	—	—	—
Thessaly	28/7/2010		10/9/2010	Negative	16/7/2011	26/7/2011	28/6/2011	13/7/2011	—	—	11/5/2012	11/5/2012	—	—	—	—
Ionian Islands	—	—	—	—	—	—	—	—	12/8/2012	29/8/2012	—	—	9/8/2013	27/8/2013	—	—
Central Macedonia	6/7/2010	5/8/2010	10/8/2010	8/9/2010	18/7/2011	29/7/2012	20/4/2011	23/6/2011	17/7/2012	24/7/2012	23/5/2012	1/6/2012	2/7/2013	15/7/2013	4/6/2013	29/7/2013
Crete	—	—	—	—	—	—	—	—	—	—	—	—	—	—	—	—
Central Greece	—	—	15/9/2010	Negative	12/8/2011	19/8/2012	21/6/2011	14/7/2011	9/7/2012	19/7/2012	15/5/2012	15/5/2012	—	—	19/6/2013	Negative
Peloponnese	—	—	—	—	—	—	1/6/2011	Negative	—	—	11/6/2012	Negative	—	—	8/5/2013	Negative

**Table 3:** Distribution of *Cx. pipiens* & *Oc. caspius* WNV pools in 2012.

Region	Identified mosquito species	Number of tested pools	Number of positive for WNV pools (% abundance)
Attica	*Cx. pipiens*	182	18 (9.9)
East Macedonia & Thrace	*Cx. pipiens*	178	22 (12.4)
Central Macedonia	*Oc. caspius*	119	13 (10.9)
Central Macedonia	*Cx. pipiens*	114	5 (4.4)
Thessaly	*Cx. pipiens*	119	13 (10.9)
Western Greece	*Cx. pipiens*	17	9 (52.9)
Central Greece	*Cx. pipiens*	53	31 (58.5)
		Total: 782	Total: 111

**Table 4:** MIR and MLE values calculated for regions studied, for years 2010–2013.

Year	Region	Mosquito species	Number of pools	Number of positive pools	Number of individuals	MIR infection rate	MLE infection rate
2010	Central Macedonia	*Culex pipiens*	110	2	6597	0.30 (CI: 0–0.01)	0.31 (CI: 0.05–1.01)

2011	Central Macedonia	*Culex pipiens*	642	42	49348	0.85 (CI: 0.59–1.11)	0.88 (CI: 0.65–1.18)
	Central Greece & Thessaly	*Culex pipiens*	99	11	2961	3.71 (CI: 1.52–5.91)	3.87 (CI: 6.66–2.08)
	East Macedonia and Thrace	*Culex pipiens*	83	7	5162	1.36 (CI: 0.35–2.36)	1.38 (CI: 0.62–2.69)
		*Ochlerotatus caspius*	73	11	12473	0.88 (CI: 0.36–1.40)	0.94 (CI: 0.50–1.62)

2012	Attica	*Culex pipiens*	182	18	2354	7.65 (CI: 4.13–11.17)	8.05 (CI: 5.08–12.24)
	East Macedonia and Thrace	*Culex pipiens*	178	22	8385	2.62 (CI: 1.53–3.72)	1.84 (CI: 2.84–4.22)
	Central Macedonia	*Culex pipiens*	119	13	62081	1.05 (CI: 0.79–1.30)	1.09 (CI: 0.85–1.38)
		*Ochlerotatus caspius*	114	5	9099	0.55 (CI: 0.07–1.03)	0.56 (CI: 0.21–1.22)
	Thessaly	*Culex pipiens*	119	13	5687	2.29 (CI: 1.04–3.53)	2.41 (CI: 1.37–3.99)
	West Greece	*Culex pipiens*	17	9	2014	4.46 (CI: 0.00–0.01)	0.01 (CI: 0.00–0.01)
	Central Greece	*Culex pipiens*	53	31	2070	14.98 (CI: 9.74–20.21)	22.80 (CI: 17.18–31.39)

2013	Attica	*Culex pipiens*	130	44	3906	10.75 (CI: 7.52–13.99)	15.76 (CI: 11.66–20.65)
